# Editorial: Mechanisms Underlying Experience-Dependent Plasticity of Cortical Circuits

**DOI:** 10.3389/fncel.2021.687297

**Published:** 2021-04-30

**Authors:** Lang Wang, Melissa L. Caras, Theofanis Karayannis, Robert C. Froemke

**Affiliations:** ^1^Interdisciplinary Institute of Neuroscience and Technology, Zhejiang University School of Medicine, Hangzhou, China; ^2^Department of Biology, University of Maryland College Park, College Park, MD, United States; ^3^Brain Research Institute of the University of Zurich, Zurich, Switzerland; ^4^Skirball Institute, Neuroscience Institute, Department of Otolaryngology, New York University Grossmna School of Medicine, New York, NY, United States

**Keywords:** cortical circuits, plasticity, experience, excitatory synapse, inhibitory synapse

Experience-dependent plasticity of the central nervous system generally occurs in response to changes of environmental stimuli. Mechanisms of experience-dependent plasticity have been traditionally studied in sensory cortices, mostly focusing on how sensory input regulates synaptic modifications and reorganization of neural circuits, as well as how the rewiring of neural circuits impacts different aspects of cortical function. Although there do seem to be some basic rules for cortical circuit refinement such as the concept of “critical period” and the involvement of inhibitory synapses, many questions are still open concerning mechanisms and functions for multiple forms of experience-dependent plasticity throughout the nervous system and across species. This Research Topic collected several original articles to provide interesting new findings and perspectives in this field.

How visual recognition memory forms remains an open question. Despite major advances in computer vision analogs of this process, the neurobiological cellular and circuit mechanisms are not fully understood. Daily exposure of awake mice to a phase-reversing visual grating stimulus leads to an enhancement of the visual-evoked potentials (VEPs) in layer 4 of the primary visual cortex (V1) (Frenkel et al., 2006), which is a phenomenon known as stimulus-selective response potentiation (SRP). To some extent, SRP reflects the formation of a long-term visual memory for the exposed stimulus. Kim et al. used *in vivo* calcium imaging to monitor cellular activity across days during the introduction of SRP. They observed a robust depression and altered dynamics of somatic responses across days as the oriented stimulus became familiar, which is opposite to the augmented dendritic responses recorded with VEPs. These findings suggest that inhibition in V1 is strongly recruited by the augmentation of synaptic responses to suppress cellular activity, thus providing a new mechanistic basis for the formation of long-term visual recognition memory.

Although it has long been appreciated that synaptic plasticity guides development of neural circuits in early postnatal life, of course there is extensive plasticity occurring in adulthood as well. Based on the cellular and circuit mechanisms recently found in visual and auditory systems, Ribic et al. summarized the similarities and differences of experience-dependent plasticity between the developing and adult brain. A primary finding is that passive exposure to environmental stimuli is sufficient to drive robust plasticity in early life, whereas active learning during task performance is necessary for adult plasticity. Despite these differences, inhibitory circuits have been demonstrated to shape the plasticity in both the developing and adult brain. In the final part of this review, the author discussed distinct mechanisms for maladaptive plasticity in neurodevelopmental disorders such as autism spectrum disorders (ASDs), and suggested that understanding the mechanisms of active learning in youth will be important for non-invasive therapies for the recovery of cortical function in patients with these conditions.

As one of the classic models of experience-dependent plasticity, ocular dominance plasticity (ODP) during the critical period for visual cortical development was first described by Wiesel and Hubel in cat V1 (Wiesel and Hubel, 1963a,b). Aiming at a better understanding of the mechanisms for critical period plasticity, Xu et al. proposed a new model with two distinct types of excitatory synapses: innate synapses which establish basic networks with innate cortical function, and gestalt synapses which govern experience-dependent refinement of cortical circuits. The authors suggested that the cooperation of two postsynaptic scaffolding proteins, PSD-93 and PSD-95, determines the maturation of gestalt synapses. With this model, the authors brought different gestalt synapse-based mechanisms for critical period ODP and adult ODP. Investigating the mechanisms for distinct forms of critical period plasticity is fundamental for understanding how the normal function of cortical circuits is achieved, which will benefit the development of therapeutics to correct defects of cortical circuit refinement at both young and adult stages.

Emerging new techniques such as RNA-seq and proteomic analysis now make it feasible to quantify large numbers of genes and proteins related to experience-dependent plasticity. These big data sets raise challenges for understanding the diversity of molecular mechanisms governing experience-dependent plasticity during development and adulthood. By using high-dimensional data analysis to identify collections of plasticity-related proteins in V1 of rat, cat, and human, Balsor et al. provided a primer for constructing a plasticity phenotype and then using this analytical framework to identify the changes that accompany different types of visual experience. This approach helps reveal subtle aspects of sensory cortical development, which holds potential for addressing clinically-relevant issues in sensory development and dysfunction. Importantly, all the R code used in this paper is available to the community as an open-source.

There are multiple developmental time windows in which cortical circuits are susceptible to intrinsic genetic factors and extrinsic environmental stimuli during development. This has led to different notions of “critical periods” and “sensitive periods.” To differentiate “critical periods” from “sensitive periods,” Dehorter and Del Pino et al. proposed a principle based on their ultimate impact on brain structure and function. In this context, the authors re-examined the criteria that defines critical periods and summarized recently-reported mechanisms of developmental plasticity in health and disease. They further discussed the therapeutic potential of manipulating critical periods to shift developmental trajectories to restore specific aspects of brain function in several neurodevelopmental disorders. Finally, the authors surveyed recent studies reconstructing developmental trajectories of human cortical circuits *in vitro* by using stem cell technologies such as 3D brain organoids, and highlighted the possibility of taking advantages of these cutting-edge techniques to find new clinical strategies for treating neurodevelopmental disorders.

It has been widely reported that visual experience promotes the maturation of inhibitory synapses from parvalbumin-positive interneurons (PV-INs) onto excitatory pyramidal neurons in V1 during postnatal development (Morales et al., 2002; Griffen and Maffei, 2014). However, the molecular and cellular mechanisms underlying this developmental process are not fully understood. By using dual-patch recordings to precisely quantify the connections from PV-INs onto pyramidal neurons, Huang and Kirkwood et al. observed significant increases in both connection probability and the number of synaptic release sites during postnatal development, thus contributing to an enhancement of inhibitory synaptic strength. Dark rearing from eye-opening prevented the increase of release site number, which was rescued by brief light re-exposure, suggesting the maturation of PV-INs synapses heavily relies on visual experience. Moreover, the authors found that agonists or antagonists of cannabinoid-1 (CB1) receptors, respectively, mimicked or blocked the effect of light re-exposure on reduced synaptic release sites induced by visual deprivation. This study reveals a gradual increase of both synaptic connectivity and strength of PV-INs during postnatal development, and indicates an important role of cannabinoid signaling for experience-dependent maturation of PV-INs synapses.

Fast learning is a behavior related to long-term memory formation after a single and brief experience, and is a core principle of human episodic memory. Piette et al. reviewed the synaptic and neuronal mechanisms underlying fast learning. They first described the variety of behavioral paradigms used to probe fast learning memories. Then they discussed neuronal activity patterns including sparse and burst firing which are initiated after the presentation of a single or brief stimulus. The authors further summarized the accumulating evidence on changes of fast learning-induced structural, synaptic and intrinsic plasticity. The mechanisms discussed in this review help uncover the cellular and synaptic-basis of fast learning rules, and also inform the development of biologically interpretable models of fast learning which might be applicable to machine learning.

Together, the articles included in this Research Topic describe novel mechanisms, new computational models, and refined hypotheses for experience-dependent plasticity of cortical circuits. These meaningful insights improve our understanding of the function and dysfunction of cortical circuits.

## Author Contributions

LW, MC, TK, and RF wrote and edited the manuscript. All authors contributed to the article and approved the submitted version.

## Conflict of Interest

The authors declare that the research was conducted in the absence of any commercial or financial relationships that could be construed as a potential conflict of interest.
